# Renin-angiotensin system inhibitors mitigate radiation pneumonitis by activating ACE2-angiotensin-(1–7) axis via NF-κB/MAPK pathway

**DOI:** 10.1038/s41598-023-35412-0

**Published:** 2023-05-23

**Authors:** Changsheng Cong, Shiying Niu, Yifan Jiang, Xinhui Zhang, Wang Jing, Yawen Zheng, Xiaoyue Zhang, Guohai Su, Yueying Zhang, Meili Sun

**Affiliations:** 1 Department of Oncology, Central Hospital Affiliated to Shandong First Medical University, Jinan, 250013 Shandong China; 2 Department of Oncology, Jinan Central Hospital, Shandong University, Jinan, 250013 Shandong China; 3 Shandong Provincial Hospital Affiliated to Shandong First Medical University, Jinan, 250021 Shandong China; 4 Department of Pathophysiology, Academy of Clinical and Basic Medicine, Shandong First Medical University & Shandong Academy of Medical Sciences, Jinan, 250062 Shandong China; 5 Department of Pathology, Linfen Central Hospital, Linfen, 041099 Shanxi China; 6 Department of Pathology, Shandong Medicine and Health Key Laboratory of Clinical Pathology, Shandong Lung Cancer Institute, Shandong Institute of Nephrology, The First Affiliated Hospital of Shandong First Medical University & Shandong Provincial Qianfoshan Hospital, Jinan, 250013 Shandong China; 7 Department of Cardiology, Central Hospital Affiliated to Shandong First Medical University, Jinan, 250013 Shandong China

**Keywords:** Diseases, Medical research, Oncology

## Abstract

Radiation pneumonitis (RP) affects both patients and physicians during radiation therapy for lung cancer. To date, there are no effective drugs for improving the clinical outcomes of RP. The activation of angiotensin-converting enzyme 2 (ACE2) improves experimental acute lung injury caused by severe acute respiratory syndrome coronavirus, acid inhalation, and sepsis. However, the effects and underlying mechanisms of ACE2 in RP remain unclear. Therefore, this study aimed to investigate the effects of angiotensin-converting enzyme inhibitors and angiotensin II receptor blockers on RP and ACE2/angiotensin-(1–7)/Mas receptor pathway activation. We found that radiotherapy decreased the expression of ACE2 and that overexpression of ACE2 alleviated lung injury in an RP mouse model. Moreover, captopril and valsartan restored ACE2 activation; attenuated P38, ERK, and p65 phosphorylation; and effectively mitigated RP in the mouse model. Further systematic retrospective analysis illustrated that the incidence of RP in patients using renin-angiotensin system inhibitors (RASis) was lower than that in patients not using RASis (18.2% vs. 35.8% at 3 months, *p* = 0.0497). In conclusion, the current findings demonstrate that ACE2 plays a critical role in RP and suggest that RASis may be useful potential therapeutic drugs for RP.

## Introduction

Radiotherapy is important for lung cancer in both definitive and palliative settings. Radiation-induced lung injury (RILI) is a common complication of thoracic radiotherapy. RILI manifests acutely as radiation pneumonitis (RP) and chronically as radiation pulmonary fibrosis. Although the improvements in radiation technique have led to lower rates of RP, recent data suggest that RILI incidence varies from 5 to 25%^1,2^. With the development of immunotherapy, the regimen of concurrent chemoradiation therapy (cCRT) followed by anti-programmed death ligand 1 (PD-L1) inhibitor therapy has been the current standard of care for the treatment of unresected stage III non-small cell lung cancer^3^. However, the increased incidence of RP associated with this regimen is a major dose-limiting toxicity of both thoracic radiation therapy and concurrent or sequential PD-1/PD-L1 therapies^4–7^. In the PACIFIC study, the incidence of grade 3 RP was 14.3% in the durvalumab group versus 2.5% in the observation group^8^. At present, there are no established guidelines for the treatment of RP. Glucocorticoids are recommended by most experts to treat symptomatic RP but have shown limited efficacy. Therefore, it is important to develop new therapies to mitigate RP.

Angiotensin (Ang)-converting enzyme 2 (ACE2) is highlighted by the recent COVID-19 pandemic because it is the receptor of severe acute respiratory syndrome coronavirus (SARS-CoV) and SARS-CoV-2. However, ACE2 plays an important protective role by antagonizing the activation of the classical renin-Ang system. An imbalance between the Ang-converting enzyme/Ang II/Ang II type 1 receptor (ACE/Ang II/AT1R) and ACE2/Ang-(1–7)/Mas receptor [ACE2/Ang-(1–7)/MasR] pathways and the loss of ACE2 are critical contributing factors to multisystem inflammation^9–11^, which promotes severe pneumonia and notable mortality in SARS-CoV-infected patients^10^. Increasing the activity of the ACE2/Ang-(1–7)/MasR pathway improved experimental acute lung injury caused by SARS-CoV, acid inhalation, and sepsis^12–14^.

However, the effects and underlying mechanisms of ACE2 in the RP model remain unclear. In addition, previous studies have reported that renin-Ang system inhibitors (RASis), such as Ang-converting enzyme inhibitors (ACEIs) and Ang II receptor blockers (ARBs), play protective roles by activating the ACE2/Ang-(1–7)/MasR pathway in a variety of cardiovascular diseases^15,16^. Therefore, in this study, we also examined the effects of ACEIs and ARBs on RP and ACE2/Ang-(1–7)/MasR pathway activation.

## Materials and methods

### Animals

Eight-week-old male C57BL/6 mice were obtained from the Jinan Pengyue Experimental Animal Co., Ltd. The mice were randomly divided into seven groups: control group (n = 15); irradiation group (IR group) (n = 15); IR + Adeno-associated virus (AAV)-NC group (n = 15, mice were injected with unloaded AAV via the tail vein, and chest irradiation was conducted 3 weeks after injection); IR + AAV-ACE2 group (n = 15), mice were injected with ACE2-overexpressing AAV via the tail vein, and chest irradiation was performed 3 weeks after injection); IR + AAV-ACE2 + A779 group (n = 15, mice were injected with ACE2-overexpressing AAV via the tail vein, chest irradiation was performed 3 weeks after injection, and A779 a selective MasR antagonist, was injected intraperitoneally every day after irradiation); IR + Captopril group (n = 15); and IR + Valsartan group (n = 15). The animals were kept at an environmentally controlled room temperature (22 ± 2 °C) in a 12/12 h light/dark cycle, with sterilized food and water supplied. All animal studies were approved by the Animal Care Committee of Shandong Provincial Hospital (No. 2021–632). All methods were performed in accordance with the relevant guidelines and regulations. The study was conducted according ARRIVE guidelines.

### Experimental mouse models

After anesthetization, the mice were placed in the irradiation field, and their chest was fully exposed. Single thoracic irradiation with 6 MV X-rays was performed at a dose rate of 2 Gy/min at a dose of 20 Gy, as previously described^17^. The irradiation field measured 40 cm × 2 cm. All irradiated animals were monitored for up to 5 weeks post-irradiation. AAV (2 × 10^11^ virus particles/mouse) was injected through the caudal vein 3 weeks before irradiation. A779 was orally administered 2 days before irradiation at a dose of 500 µg/kg/day^18^. Captopril was administered orally 2 days before irradiation at a dose of 50 mg/kg/day and maintained for 5 weeks after irradiation^19^. Valsartan (40 mg/kg/day) was administered orally for 5 weeks after irradiation^20^. The control group was simultaneously orally administered saline.

### Sample collection

Five weeks after irradiation, all mice were euthanized (pentobarbital overdose), and the lung tissues were resected. Histological and immunohistochemical analyses were performed on the right lung tissue. The left lung tissue was used for western blot analysis and real-time quantitative RT-PCR.

### Histologic examination

Hematoxylin and eosin staining was used to assess histological changes in the lung tissue. Briefly, the lung tissues were fixed in 10% formalin, paraffin-embedded, and then sectioned into 5-µm-thick slices (Leica, Germany). Next, the slices were dewaxed in xylene, rehydrated by exposure to graded ethanol, and stained with hematoxylin and eosin. Finally, the sections were observed under a microscope.

### Immunohistochemistry staining

The expression of IL-6 and TNF-α in lung tissues was detected by immunohistochemistry staining. Lung tissue sections were deparaffinized in xylene and dehydrated using graded alcohol concentrations. The sections were antigen-repaired using EDTA-repairing fluid for 20 min. Next, the sections were incubated with 5% BSA (AR0004, Boster, China) for 30 min at 37 °C. The slides were then incubated with primary antibodies against IL-6 (1:100, #bs-0782R, Bioss, Beijing, China) and TNF-α (1:100, #bs-10802R, Bioss, Beijing, China) at 4 °C overnight. After washing with PBS, horseradish peroxidase-labeled secondary antibody was added for 60 min at 37 °C. DAB staining was performed to visualize the slides. Two experienced pathologists performed double-blinded readings. IL-6 or TNF-α is positively expressed as brown-yellow granules on the cytoplasm and (or) nucleus. Using semi-quantitative judgment, the percentage of positive cells and intensity of staining under the microscope were scored. The frequency of positive cells was defined as follows: 0, less than 5%; 1, 5% to 25%; 2, 26% to 50%; 3, 51% to 75%; and 4, greater than 75%. The intensity was scored as follows: 0, colorless; 1, light yellow; 2, brownish yellow; and 3, brown. The staining index (values, 0–12) was determined by multiplying the staining intensity score with the frequency of positive cells. The staining index was scored as follows: 0, negative (−); 1–4, weak ( +); 5–8, moderate (+ +); and 9–12, strong (+ + +).

### Detection of lung function

Whole-body plethysmography was used to detect changes in lung function. First, the body-tracing box was connected to a sensor outside the box, and the mice were placed in a closed box for detection. When an animal breathes, the fluctuation in its chest changes the volume in the body-tracing box, and this change is converted into an electrical signal by a pressure transducer and amplifier. After being processed by a computer, the breathing curve is displayed on the computer, and the images are processed by a software to calculate respiratory parameters such as tidal volume, peak inspiratory flow rate, and peak expiratory flow rate.

### Enzyme-linked immunosorbent assay (ELISA)

Commercially available mouse ELISA kits were used to determine the levels of Ang II (CK-E21284; MLBIO, Shanghai, China) and Ang-(1–7) (CK-E20733; MLBIO, Shanghai, China) in lung tissue according to the manufacturer’s instructions. The absorbance was measured at 450 nm using a microplate reader.

### qRT-PCR

Total RNA was extracted from lung tissues using TRIzol (Vazyme Biotech Co., Nanjing, China), following the manufacturer’s instructions. The isolated RNA was reverse transcribed into cDNA according to the HiScript II Q RT SuperMix protocol for qPCR (Vazyme Biotech Co., Nanjing, China). Real-time PCR was performed using the SYBR qPCR Mix (Vazyme Biotech Co., Nanjing, China). Data were calculated using the 2^-ΔΔt^ method. Each experiment was performed in triplicate. MasR forward primer 5′-ACAACACGGGCCTCCTATCTG-3′ and reverse primer 5′-GAAGGGCACAGACGAATGCT-3′, β-actin forward primer 5′-GGCTGTATTCCCCTCCATCG-3′ and reverse primer 5′-CCAGTTGGTAACAATGCCATGT-3′.

### Western blotting

Lung tissues were homogenized in RIPA lysis buffer (Beyotime, China) with protease inhibitors, followed by centrifugation at 12,000 rpm at 4 °C, and the supernatants were collected. The proteins were separated in 8–10% sodium dodecyl sulfate-poly-acrylamide gels and transferred to polyvinylidene fluoride membranes. The membranes were cut prior to hybridization with antibodies. Next, the membranes were incubated with primary antibodies against ACE (1:1,000; #bs-0439R, Bioss, Beijing, China), ACE2 (1:1,000, #21,115–1-Ig, Proteintech, China), ATIR (1:1,000; #bs-0630R, Bioss, Beijing, China), p38 (1:500 #OM125772 OmnimAbs China), p-p38 (#bs-0636R 1:1,000 Bioss), JNK (#OM260186, 1:500, OmnimAbs), p-JNK (#bs-17591R, 1:1,000, Bioss), ERK1/2 (#OM125780, 1:500, OmnimAbs, China), p-ERK1/2 (#bs-3016R, 1:1,000, Bioss), NF-κB p65 (#66,535–1-lg, 1:1,000, PTG), and p-NF-κB p65 (#bs-3485R, 1:1,000, Bioss) at 4 °C overnight. The membranes were then incubated with horseradish peroxidase-conjugated secondary antibodies (1:5,000, #bs0295M, Bioss, Beijing, China) for 1 h at room temperature. Finally, the proteins were visualized using ECL reagents (Millipore, USA) and analyzed using ImageJ.

### Clinical data

To further verify the protective effect of RASis on RP, consecutive patients concurrently being treated with thoracic radiation for non-small cell lung cancer between October 2017 and December 2019 at our cancer center were reviewed. RP was first assessed 1 month after radiotherapy (RT) using contrast-enhanced Computed Tomography (CT) and every 2 months thereafter for up to 1 year. The diagnosis of RP should meet the following three criteria^21^: (1) pulmonary symptoms, including dyspnea or cough, or both; (2) CT-based imaging changes involving the radiated field; and (3) symptoms occurring within 12 months after the completion of radiation therapy. This case study was conducted in accordance with the ethical standards of the Declaration of Helsinki and approved by the Central Hospital Affiliated to Shandong First Medical University (No.2022–033-01). The need for informed consent was waived due to retrospective nature of the study by the Research Ethics Committee of Central Hospital Affiliated to Shandong First Medical University.

### Statistical analysis

Data were analyzed using SPSS version 25.0 (SPSS, Chicago, IL, USA). Continuous variables were presented as mean ± SEM and compared using one-way analysis of variance (ANOVA) for multiple groups. For the population study, the actuarial risk of developing RP was estimated using the Kaplan–Meier method. The RASi and no-RASi groups were compared using the log-rank test. *P* < 0.05 was considered statistically significant.

### Ethical approval

All animal studies were approved by the Animal Care Committee of Shandong Provincial Hospital (No. 2021–632). All methods were performed in accordance with the relevant guidelines and regulations. The study was conducted according ARRIVE guidelines.

This case study was conducted in accordance with the ethical standards of the Declaration of Helsinki and approved by the Central Hospital Affiliated to Shandong First Medical University (No.2022–033-01). The need for informed consent was waived due to retrospective nature of the study by the Research Ethics Committee of Central Hospital Affiliated to Shandong First Medical University.

## Results

### Overexpression of ACE2 alleviated RP

**Figure 1:**
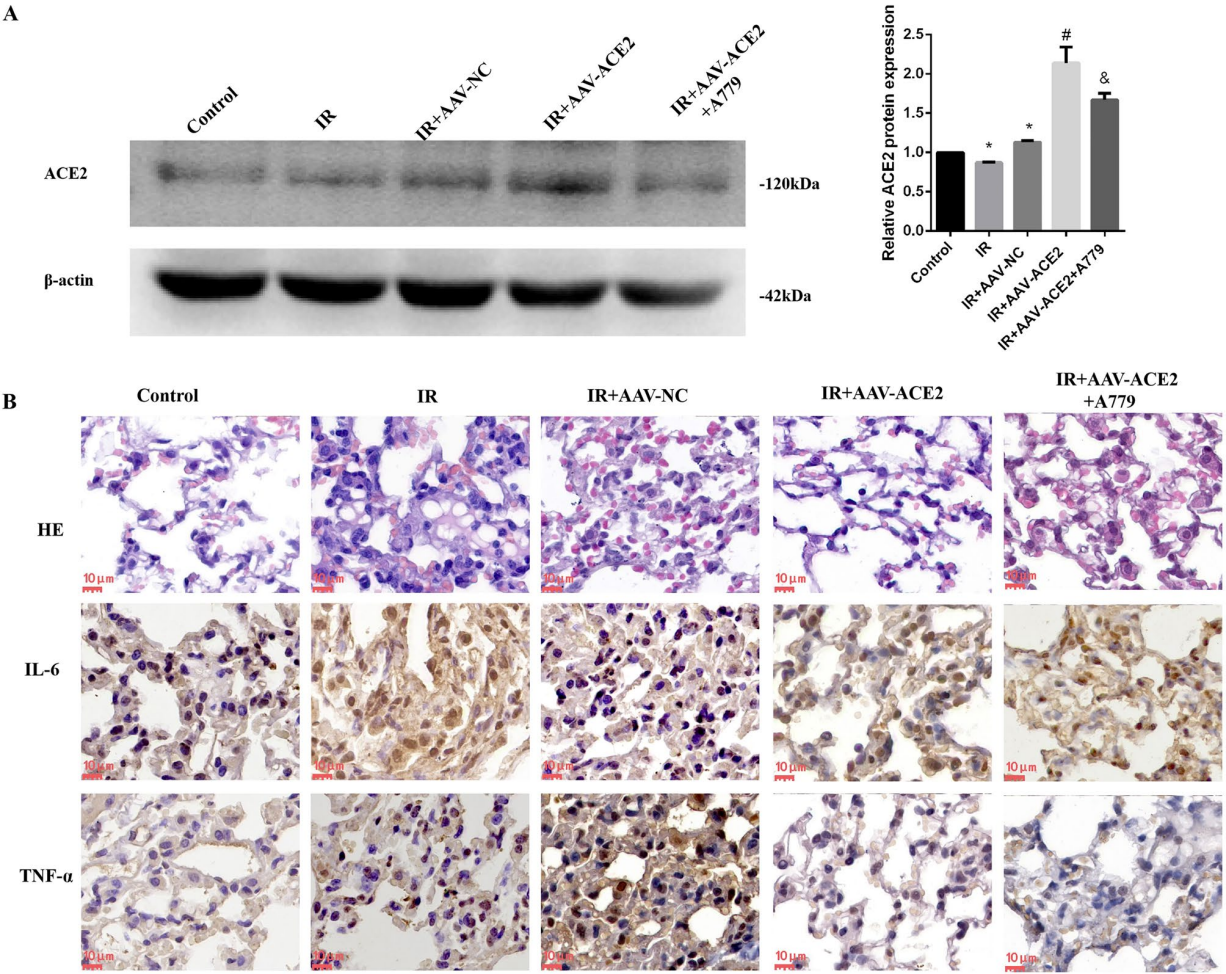
Analysis of histopathological changes and inflammatory factors (IL-6, TNF-α) in lung tissues by HE or immunohistochemical staining. A, Western blotting analysis (left) and quantification of ACE2 expression (right). B, HE, IL-6, and TNF-α staining. Scale bars, 10 μm. *Compared with the control group, *p* < 0.05. ^#^Compared with the IR group, *p* < 0.05. ^&^Compared with AAV-ACE2 group, *p* < 0.05. ACE2: angiotensin-converting enzyme 2; HE, hematoxylin and eosin; IR, irradiation.

To verify the viral transfection effect of AAV-ACE2, we evaluated the expression of ACE2 in the lung tissue by western blotting. The results showed that irradiation significantly decreased the expression of ACE2, while AAV-ACE2 treatment increased ACE2 expression (Fig. 1A). Next, we detected pathological changes in the lungs by hematoxylin and eosin staining. As shown in Fig. 1B, compared with the control group, the IR group showed destruction of the alveolar histological structure, capillary dilatation and congestion, and inflammatory cell accumulation in the lung tissues. Lung injury was markedly reduced in the AAV-ACE2 group, whereas A779 treatment attenuated the effect of ACE2 overexpression, compared with the IR group. To observe the changes in inflammatory factor expression, we used immunohistochemistry to detect the expression of IL-6 and TNF-α. The results demonstrated that the expression of IL-6 and TNF-α was markedly decreased in the AAV-ACE2 group compared with the IR group, while A779 partly reversed this trend (Fig. 1B, Table 1). As expected, ACE2 overexpression significantly alleviated RP.

**Table 1:** Score of IL-6 and TNF-α expression of Fig. 1B.

	Control	IR	IR + AAV-NC	IR + AAV-ACE2	IR + AAV-ACE2 + + A779
IL-6	1.67 ± 0.58	11 ± 1.73*	10 ± 1.73*	1.67 ± 0.58^#^	10 ± 1.73^&^
TNF-α	1.33 ± 0.58	8 ± 1.73*	11 ± 1.73*	1.33 ± 0.58^#^	7 ± 1.73^&^

*p < 0.05, compared with the control group; ^#^, *p*< 0.05, compared with the IR group. ^&^ *p* < 0.05, compared with the IR + AAV-ACE2 group.

### Overexpression of ACE2 improved lung function in RP mice

To further verify the protective effect of ACE2, we examined lung function in mice. The results showed that the lung function indices of the AAV-ACE2 group were significantly increased compared with the IR group, while A779 partly reversed this increase. Thus, ACE2 overexpression significantly improved lung function in radiation-treated mice (Table 2).

**Table 2:** Lung function parameters of C57BL/6 mice in each group.

Groups	TVb (ml)	MVb (ml/min)	PIFb (ml/s)	PEFb (ml/s)	EF50 (ml/s)
Control	0.29 ± 0.02	138.13 ± 17.87	10 ± 0.9	6.07 ± 0.77	0.4 ± 0.07
IR	0.24 ± 0.02***	107.61 ± 16.3**	8.09 ± 0.87***	4.95 ± 0.79*	0.3 ± 0.06***
AAV-NC	0.24 ± 0.02	113.04 ± 15.22	8.4 ± 0.95	5.28 ± 0.57	0.32 ± 0.04
AAV-ACE2	0.27 ± 0.01^#^	123.51 ± 8.41^#^	9 ± 0.59^#^	5.8 ± 0.72^#^	0.37 ± 0.02^#^
AAV-ACE2 + A779	0.24 ± 0.01^&&^	112.1 ± 9.05^&^	8.22 ± 0.45^&^	4.92 ± 0.57^&^	0.32 ± 0.02^&^

TVb: Tidal Volume (box); MVb: Minute Volume (box); PIFb: Peak Inspiratory Flow (box); PEFb: Peak Expiratory Flow (box); EF50: Expiratory Flow at the point 50% of TV. Compared with the control group, *indicates *p* < 0.05, **p < 0.01, ***p < 0.001. Compared with IR group, ^#^*p* < 0.05. Compared with AAV-ACE2 group, ^&^ indicates *p* < 0.05, ^&&^*p* < 0.01.

### Overexpression of ACE2 mediated activation of ACE2/Ang-(1–7) axis.

**Figure 2:**
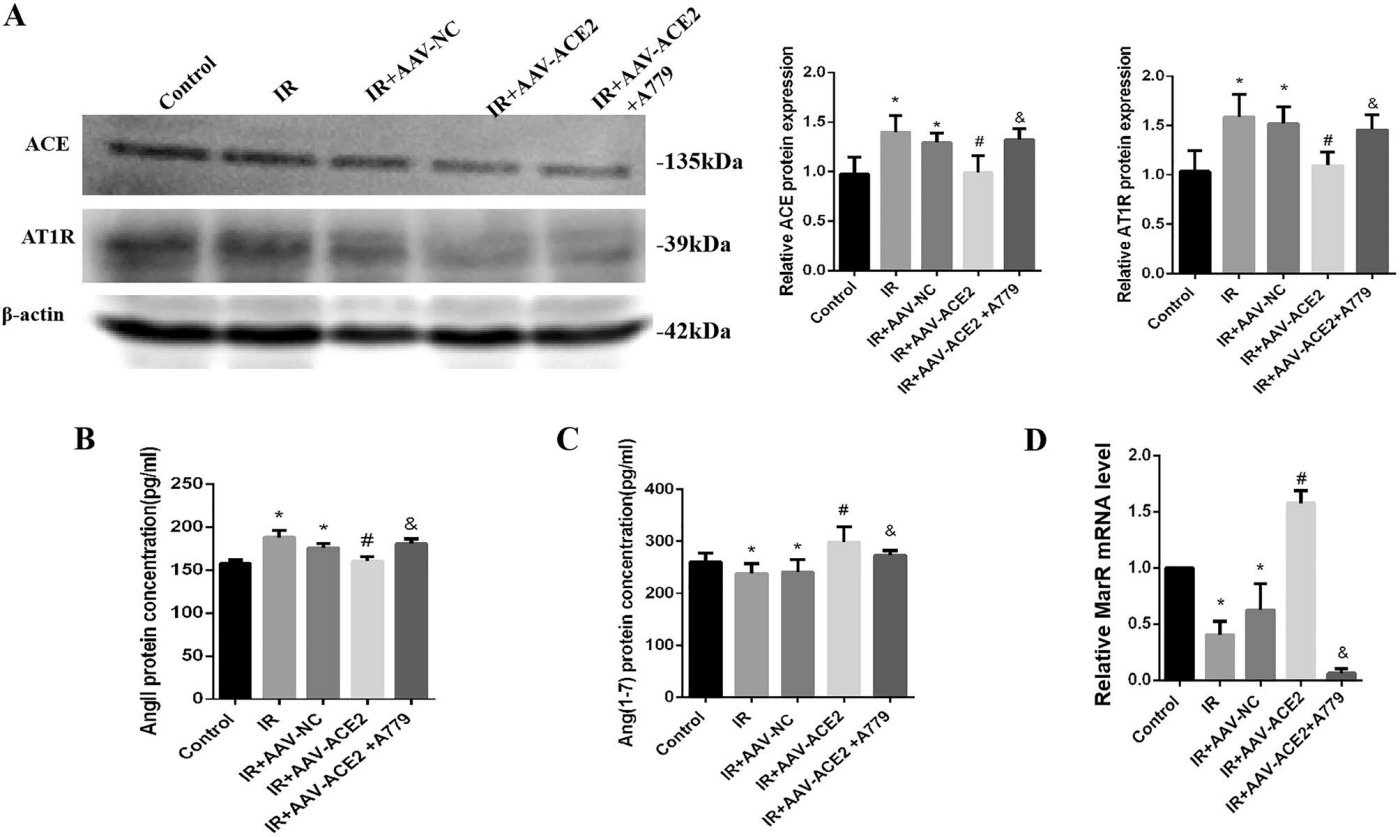
Overexpression of ACE2 mediated activation of ACE2/Ang-(1–7) axis. (**A**) Western blotting analysis (left) and quantification of the expression of ACE2 (middle histogram) and AT1R (right histogram) in the lung tissue. (**B**, **C**) Ang II and Ang-(1–7) levels in lung tissues were detected using enzyme-linked immunosorbent assay. **D,** mRNA levels of MasR in the lung tissue. *Compared with the control group, *p* < 0.05, **p < 0.01. ***p < 0.001. ^#^Compared with the IR group, *p* < 0.05, ^##^*p* < 0.01. ^&^Compared with AAV-ACE2 group, *p* < 0.05, ^&&^* p* < 0.01. ACE2: angiotensin-converting enzyme 2; Ang, angiotensin; AT1R, angiotensin II type 1 receptor; IR, irradiation; MasR, Mas receptor.

The balance between the ACE/Ang II/AT1R and ACE2/Ang-(1–7)/MasR axes is critical for maintaining normal physiological functions. However, little is known about the changes that occur during RILI. The ELISA results showed that, compared with the control group, irradiation markedly increased the concentration of Ang II while decreasing the concentration of Ang-(1–7) in the lung tissues. However, the concentration of Ang II decreased and the concentration of Ang-(1–7) was obviously increased in the AAV-ACE2 group, whereas the A779 treatment reversed this change. In addition, we detected the expression of ACE, ACE2, AT1R, and MasR using western blotting or qPCR. The expression of ACE and AT1R was markedly upregulated under irradiation but downregulated in the AAV-ACE2 group compared with the control group. Additionally, MasR expression was downregulated in the IR group, while AAV-ACE2 treatment upregulated MasR expression (Fig. 2).

### Captopril and valsartan mitigated RP

**Figure 3:**
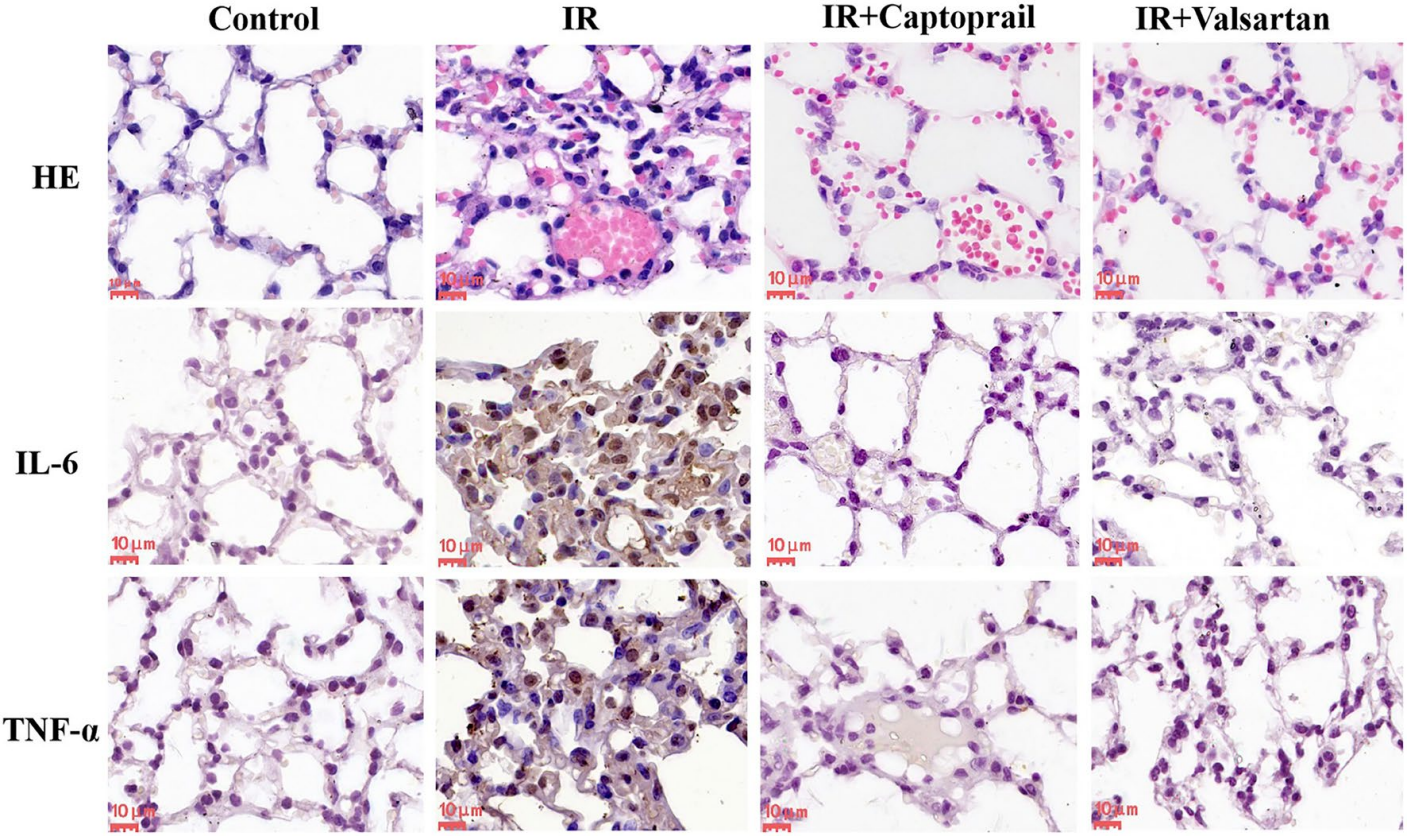
Analysis of histopathological changes and inflammatory factors (IL-6, TNF-α) in lung tissues by HE or immunohistochemical staining. Scale bars, 10 μm. IR, irradiation; HE, hematoxylin and eosin.

Compared with the control group, the irradiation group showed obvious destruction of the alveolar histological structure, capillary dilatation and congestion, and inflammatory cell accumulation in the lung tissues. Lung injury was markedly reduced after treatment with captopril and valsartan. In addition, compared with the irradiation group, captopril and valsartan treatment considerably decreased the expression of IL-6 and TNF-α (Fig. 3, Table 3). As expected, captopril and valsartan significantly improved the RP.

**Table 3:** Score of IL-6 and TNF-α expression of Fig. 3.

	Control	IR	IR + Captopril	IR + Valsartan
IL-6	0.67 ± 0.58	11 ± 1.73*	1.00 ± 0.58^#^	1 ± 0.58^#^
TNF-α	1.00 ± 0.58	10 ± 1.73*	1.33 ± 0.58^#^	0.67 ± 0.58^#^

*p < 0.05, compared with the control group; ^#^, *p*< 0.05, compared with the IR group.

### Captopril and valsartan mediated activation of ACE2/Ang-(1–7) axis

**Figure 4:**
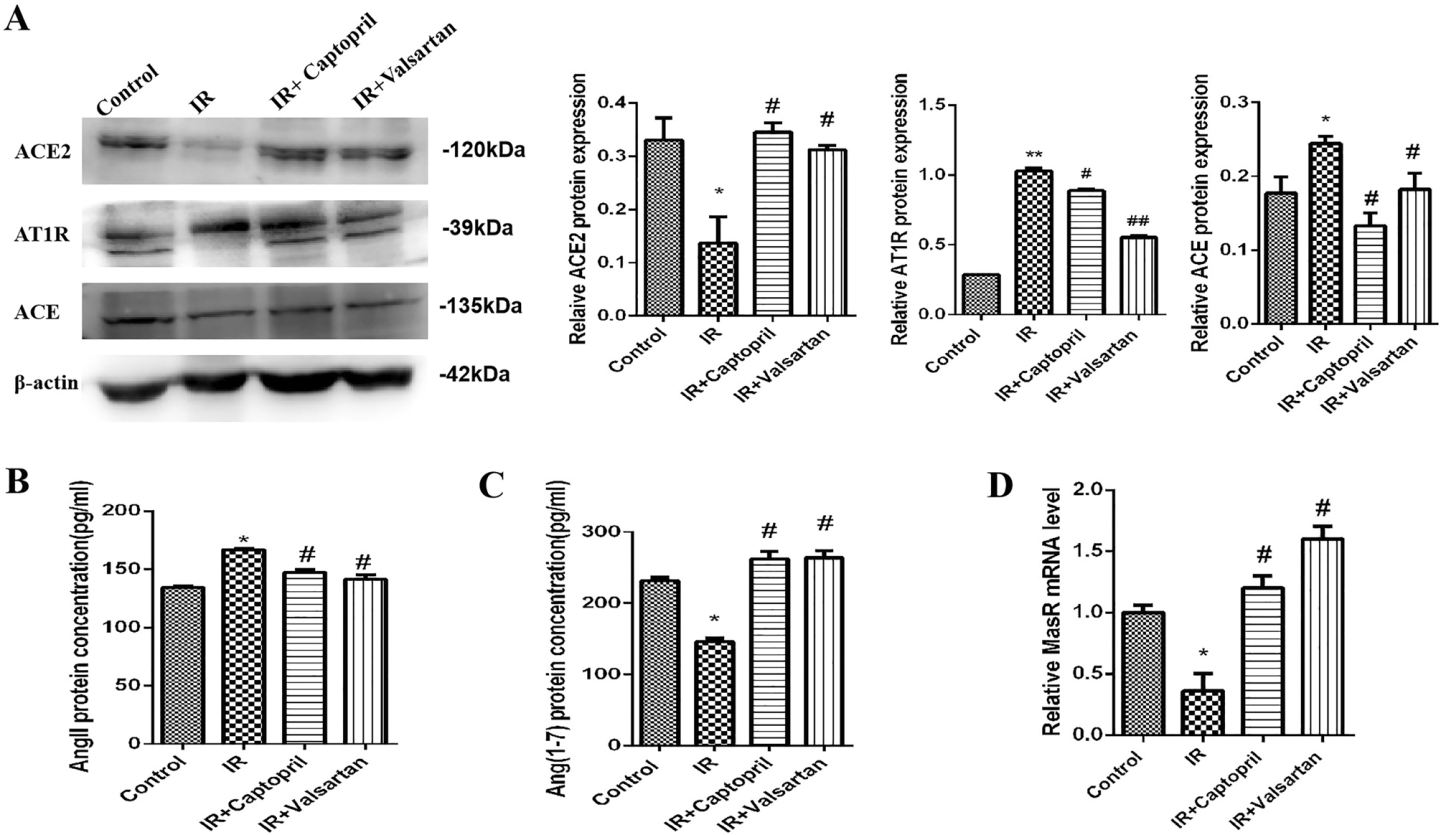
Captopril and valsartan mediated activation of ACE2/Ang-(1–7) axis. (**A**) Expression levels of ACE2, AT1R, and ACE in the lung tissue. (**B**) Levels of Ang II in the lung tissue. (**C**) Levels of Ang-(1–7) in the lung tissue. (**D**) mRNA levels of MasR in the lung tissue. *Compared with the control group, *p* < 0.05. ^#^Compared with the IR group, *p* < 0.05, ^##^* p* < 0.01. ACE: angiotensin-converting enzyme; ACE2: angiotensin-converting enzyme 2; Ang, angiotensin; AT1R, angiotensin II type 1 receptor; IR, irradiation; MasR, Mas receptor.

To explore the protective mechanisms of captopril and valsartan in RP, we evaluated the ACE2/Ang-(1–7) axis changes. First, we detected the expression of ACE, ACE2, AT1R, and MasR using western blotting and qPCR. ACE and AT1R expression was markedly upregulated under irradiation but downregulated in the captopril and valsartan group compared with the control group. In addition, the expression of ACE2 and MasR in the irradiation group was obviously downregulated compared with the control group, while captopril and valsartan reversed this change (Fig. 4A). Furthermore, the concentrations of Ang II and Ang-(1–7) in the lung tissue were measured using ELISA. Compared with the control group, irradiation noticeably increased the concentration of Ang II but decreased the concentration of Ang-(1–7) in the lung tissue. However, captopril and valsartan decreased the concentration of Ang II and increased the concentration of Ang-(1–7) (Fig. 4B–D). These results suggest that captopril and valsartan attenuate RP via ACE2/Ang-(1–7) axis activation.

### Captopril and valsartan reduce inflammatory response by inhibiting MAPK and NF-κB pathways

**Figure 5:**
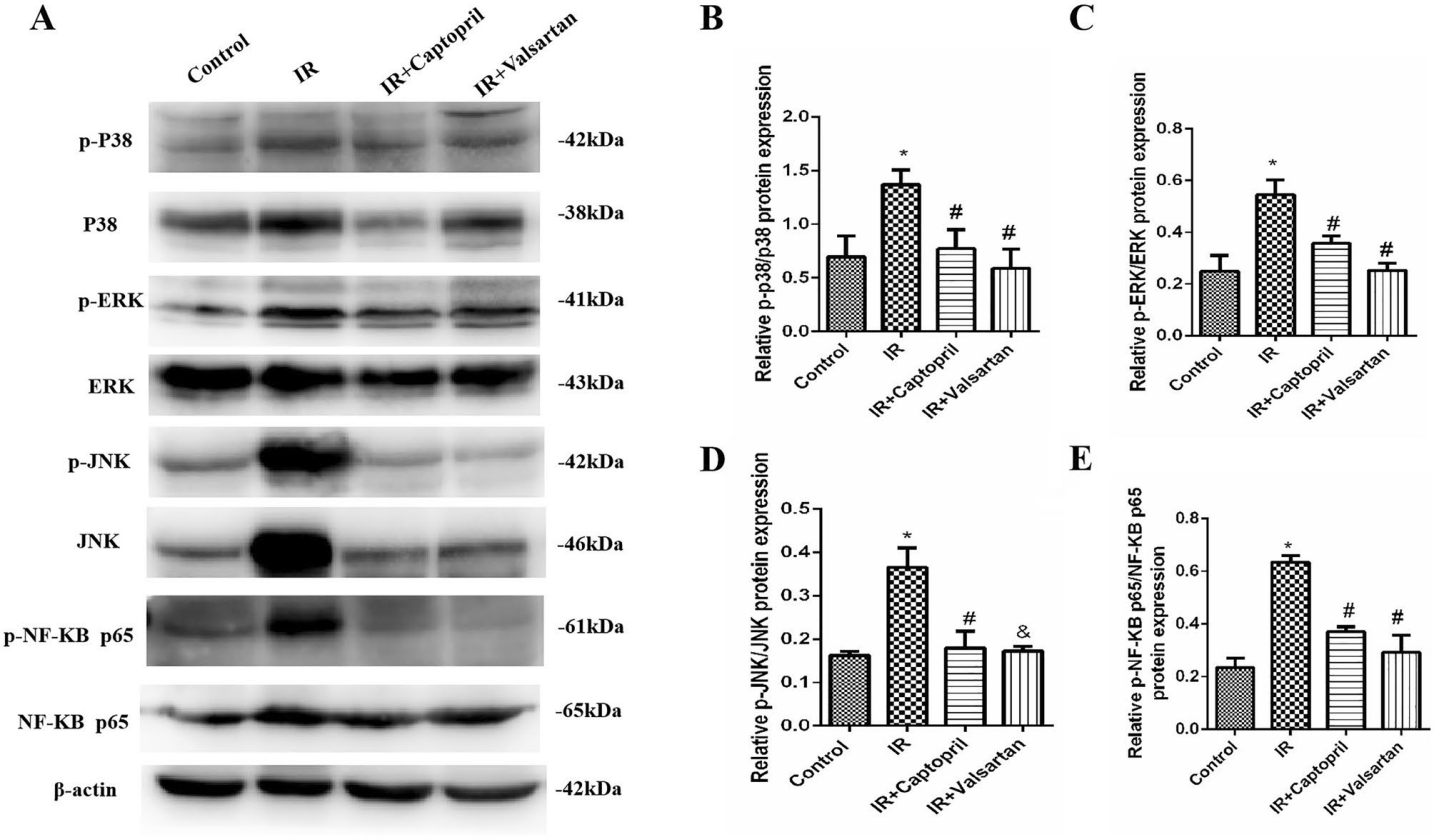
Captopril and valsartan inhibited MAPK and NF-κB pathways. (**A**) Expression of MAPKs and NF-κB in the lung tissues. (**B**) Quantitative analysis of p-p38. (**C**) Quantitative analysis of p-ERK. (**D**) Quantitative analysis of p-JNK. (**E**) Quantitative analysis of p-NF-κB p65. Data are represented as the mean ± SEM of three independent experiments. *Compared with the control group, *p* < 0.05. ^#^Compared with the IR group, *p* < 0.05. p-p38, phosphorylated p38; p-ERK, phosphorylated ERK; p-JNK, phosphorylated JNK; p-NF-κB p65, phosphorylated NF-κB p65; IR, irradiation.

We further explored the possible mechanisms of captopril- and valsartan-induced ACE2/Ang-(1–7) axis activation during IR. MAPK and p65 levels in the lung tissue were detected by western blotting. Compared to the control group, irradiation promoted the phosphorylation of p38, ERK1/2, JNK, and p65, whereas captopril and valsartan inhibited p38, ERK1/2, and JNK activation. Therefore, captopril and valsartan may attenuate RP by inhibiting the MAPK and NF-κB pathways (Fig. 5).

### RP incidence in the RASi group was lower than that in the no-RASi group

**Figure 6:**
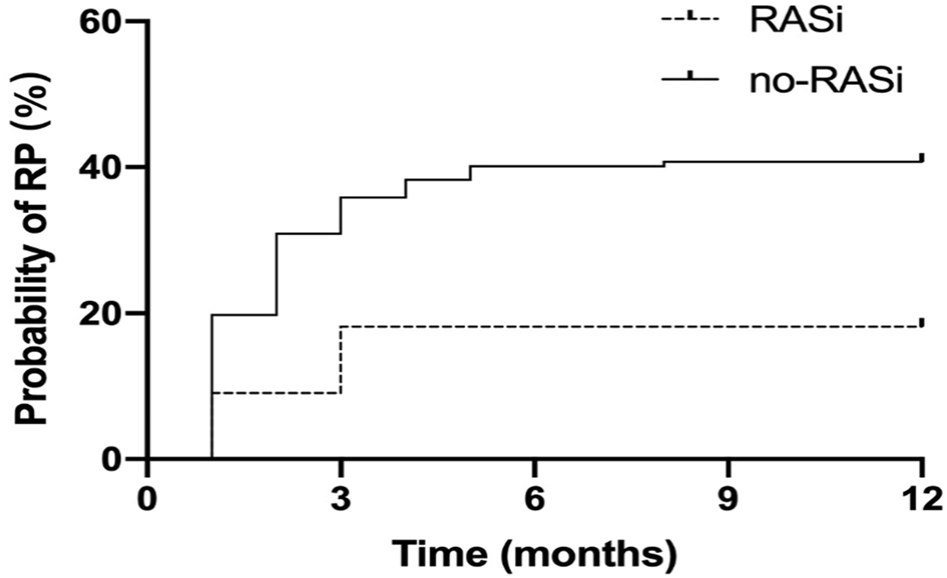
Probability of RP in RASi and no-RASi groups. RP, radiation pneumonitis; RASi, renin-angiotensin system inhibitor.

In total, 184 NSCLC patients with stage III and IV who received thoracic radiation at the primary site or had metastatic lymph nodes were enrolled; of them, 22 patients took RASis and 162 did not. The RT dose ranged from 50 to 66 Gy (IQR: 56–60), with a median dose of 60 Gy. Patients taking RASis were older and had a history of hypertension. Except for age and history of hypertension, clinical characteristics were balanced between the RASi and no-RASi groups (Table 4). A total of 70 patients (70/184, 38.0%) developed RP: 4 (18.2%) in the RASi group and 66 (40.7%) in the no-RASi group (*p* = 0.041). The time to RP development ranged from 1 to 12 months (IQR: 2–12). The actuarial risk of developing RP at 3 and 12 months was 18.2% and 18.2%, respectively, in the RASi group, compared to 35.8% and 40.7% in the no-RASi group, respectively (hazard ratio [HR] = 0.48, 95%CI 0.23–0.99, *p* = 0.0497) (Fig. 6).

**Table 4:** The characteristic of patients receiving thoracic radiotherapy.

	Variables	Total n = 184	Non-RASi n = 162(%)	RASi n = 22(%)	x^2^	*P* value
Age	mean ± SD	62.9 ± 9.10	62.3 ± 8.9	67.3 ± 9.2		0.024
Sex					0.01	0.915
	male	149	131(80.9)	18(81.8)		
	female	35	31(19.1)	4(18.2)		
ECOG score					0.02	0.898
	0	98	86(53.1)	12(54.5)		
	≥ 1	86	76(46.9)	10(45.4)		
History of smoke					0.28	0.599
	Yes	118	105(64.8)	13(59.1)		
	No	66	57(35.2)	9(40.9)		
Hypertension					43.63	< 0.001
	Yes	67	45(27.8)	22(100)		
	No	117	117(72.2)	0(0)		
Combined therapy					0.59	0.442
	Yes	106	95(58.6)	11(50.0)		
	No	78	67(41.4)	11(50.0)		
Dmean	mean ± SD	11.82 ± 3.06	11.86 ± 3.09	11.53 ± 2.86		0.635
V5					0.24	0.623
	< 60%	171	150(92.6)	21(95.5)		
	≥ 60%	13	12(7.4)	1(4.5)		
V20					0.43	0.510
	< 30%	169	148 (91.3)	21(95.5)		
	≥ 30%	15	14(8.6)	1(4.5)

## Discussion

The population at risk of RP is considerably large because most patients with thoracic cancer are expected to undergo RT in their lifetime. Treatment of acute pneumonitis depends on clinical severity and typically responds completely to corticosteroids. Numerous trials have been conducted to identify novel treatments for RP. Amifostine, a free radical scavenger, is the only radioprotective agent approved by the US Food and Drug Administration for clinical use because it has been shown to significantly reduce the rate of grade 2 or higher pneumonitis in patients receiving radiation for advanced-stage lung cancer^22^. However, the use of amifostine is limited owing to its significant side effects (i.e., hypotension and severe nausea) and poor tolerability (especially when administered intravenously)^21^. Therefore, it is important to identify new therapeutic targets and drugs for the treatment of RP. In our current study, we found that (1) ACE2 overexpression attenuated RP in a mouse model, and (2) captopril and losartan mitigated RP and activated ACE2/Ang-(1–7)/MasR axis via inhibiting the NF-κB and MAPK pathways.

ACE2 is widely expressed in lung alveolar epithelial cells and arterial and venous endothelial cells^23^. ACE2 deficiency aggravated lung injury in SARS-CoV-infected mice, showing severe acute respiratory distress syndrome/acute lung injury pathology, increased vascular permeability, increased pulmonary edema, neutrophil accumulation, and deterioration of lung function, compared to normal wild-type control mice^13^. A pilot clinical trial of recombinant human ACE2 in acute respiratory distress syndrome demonstrated that ACE2 decreased IL-6 concentrations, suggesting an anti-inflammatory effect of ACE2^26^. ACE2 converts Ang I into Ang-(1–9) and Ang II to the vasoprotective heptapeptide Ang-(1–7)^24^. Ang-(1–7) acts through MasR to counterbalance the detrimental effects of Ang II signaling, such as vasoconstriction, inflammation, cell proliferation, hypertrophy, fibrosis, and tissue remodeling^25^.

Histopathological lung injuries associated with RP were similar to virus pneumonias, including destruction of the alveolar structure, capillary dilatation and congestion, and inflammatory cell accumulation. However, the expression and the role of ACE2/Ang-(1–7)/MasR were not clear in RP. Our study showed the expression levels of ACE2, Ang-(1–7), and MasR were significantly downregulated in the RP mice, and overexpression of ACE2 significantly alleviated histopathological lung injuries and relieved the symptoms of RP. These results prompted that ACE2 plays an important role in RP. Then, further research on ACE2/Ang-(1–7)/MasR was conducted in RP mice models.

Ang-(1–7) protects against lung infection caused by influenza A virus and reduces the lethality rate by combining with MasR in a mouse model; these protective effects disappeared in MasR-deficient (MasR^-/-^) mice^27^. In contrast to the influenza A virus infection mouse model, MasR expression was reduced in the RP mouse group and upregulated in the AAV-ACE2 group. The use of A779, a specific MasR blocker, reversed the protective effects of ACE2 in the RP model, indicating that the protective effect of ACE2 in RP was partly mediated by Ang-(1–7). Altogether, these results show that the activation of the ACE2/Ang-(1–7)/MasR axis is important for alleviating lung injuries caused by RP by inhibiting inflammation.

RASis are commonly used to treat hypertension, myocardial infarction, and heart failure. A previous study found that lipopolysaccharides induced acute lung injury and decreased ACE2 expression, whereas ACEI and ARB treatment alleviated lipopolysaccharide-induced pneumonic injury^28^. Thus, ACEIs and ARBs, such as captopril and valsartan, might also inhibit the activation of ACE/Ang II/AT1R and maintain the balance between ACE/Ang II/AT1R and ACE2/Ang-(1–7)/MasR axis to alleviate RP. This study showed that the continuous use of captopril or valsartan for 5 weeks notably reduced lung injury after X-ray irradiation and greatly decreased the secretion of IL-6 and TNF-α. Both captopril and valsartan suppressed the activation of ACE/Ang II/AT1R and reversed the downregulation of the ACE2/Ang-(1–7)/MasR axis in the RP mouse model. NF-κB and MAPK activation-induced acute inflammation play an important role in acute lung injury^29^. In this study, the irradiation-induced activation of p38, ERK1/2, JNK, and p65 was inhibited by captopril and valsartan, suggesting that captopril and valsartan mitigated acute lung inflammation by inhibiting NF-κB/MAPK signaling pathways in RP.

These interesting findings prompted us further to observe the real-world effects of RASis on RP. RASis, such as captopril and valsartan, reduce the risk of cardiovascular death or hospitalization for heart failure and improve symptoms in patients with chronic heart failure with reduced ejection fraction^30,31^. A systematic retrospective analysis found that the rate of grade 2 or higher pneumonitis was 2% in ACEI users and 11% in non-ACEI after cCRT^32^. Another study found that using RASis might alleviate RP in patients with lung cancer after stereotactic body radiotherapy^33^. In this study, we found that the incidence of RP in the RASi group was significantly lower than that in the no-RASi group during the 12 months after the completion of radiation therapy.

However, this study had some limitations. In the current study, we did not knockdown or knockout ACE2, test whether RASis can mitigate IR, or assess the effect of RASis on MAPK and inflammatory signaling pathways; hence, it is impossible for us to draw a definite conclusion. Further studies using an ACE2-knockout model may resolve this issue.

Activation of the ACE2/Ang-(1–7)/MasR axis may play an important protective role in the RP mouse model. Captopril and valsartan have shown promising benefits for RP in both mouse models and retrospective observational cohort studies. Thus, RASis may be potential therapeutic drugs for RP.

## Supplementary Information

Supplementary Information.

## Data Availability

The original contributions presented in this study are included in the article, further inquiries can be directed to the corresponding author.

## Abbreviations

RP: Radiation pneumonitis
ACE2: Angiotensin-converting enzyme 2
RASi: Renin-angiotensin system inhibitors
RILI: Radiation-induced lung injury
PD-L1: Anti-programmed death ligand 1
ACEI: Angiotensin-converting enzyme inhibitor
AT1R: Angiotensin II type 1 receptor
Ang II: Angiotensin II
Ang-(1–7): Angiotensin-(1–7)
